# Kings Nursing Fathers

**Published:** 1874-01

**Authors:** 


### KINGS NURSING FATHERS.

THE Scriptures promised of old that the time would come when "Kings should be the nursing fathers" of the church, meaning thereby that men of position, and wealth, and power would eventually become the friends of Christianity, and would consecrate themselves, their money and their influence to its promotion. Among the money kings of the United States, who have of late years given their tens and hundreds of thousands, and even millions to advance the highest and best interests of humanity, such as Peabody, and Cooper, and Roberts, and Vanderbilt, and Drew, there should be placed another name that has many a time before underwritten its thousands upon thousands, that is, James Brown, who is at the head of the house known over the civilized world, and very few are as well known as that of "Brown Brothers,"of New York—he has lately given three hundred thousand dollars to the "Union Theological Seminary," the interest of which is to be appropriated to the purpose of aiding young men of talents and piety to become ministers of the Gospel. The multitudes who have patronized that old and honorable house, in procuring letters of credit on going abroad, will feel gratified to know that the "profits" of the "transaction" have been laid up in so large a part for so glorious a purpose, and the silent, and earnest, and spontaneous ejaculations of the good will surely go up to the Mighty One that the choicest of earthly blessings, and of heavenly grace, should be meted out to the eminent banker, who must be now nearly his fourscore, who is wise enough to give while he lives, seeing the "time is short and the night cometh, when no man can work." May every reader in his sphere "go and do likewise."

